# Acute exacerbation of fibrotic hypersensitivity pneumonitis: incidence and outcomes

**DOI:** 10.1186/s12931-021-01748-2

**Published:** 2021-05-20

**Authors:** Jieun Kang, Yeon Joo Kim, Jooae Choe, Eun Jin Chae, Jin Woo Song

**Affiliations:** 1 Department of Pulmonary and Critical Care Medicine, Asan Medical Center, University of Ulsan College of Medicine, 88 Olympic-Ro 43-Gil, Songpa-gu, Seoul, 05505 Republic of Korea; 2 Division of Pulmonary and Critical Care Medicine, Department of Internal Medicine, Ilsan Paik Hospital, Inje University College of Medicine, Goyang-si, Gyeonggi-do Republic of Korea; 3 Department of Radiology and Research Institute of Radiology, Asan Medical Center, University of Ulsan College of Medicine, Seoul, Republic of Korea

**Keywords:** Hypersensitivity pneumonitis, Acute exacerbation, Incidence, Outcome

## Abstract

**Background:**

Patients with fibrotic hypersensitivity pneumonitis (HP) show variable clinical courses, and some experience rapid deterioration (RD), including acute exacerbation (AE). However, little is known about AE in fibrotic HP. Here, we retrospectively examined the incidence, risk factors, and outcomes of AE in fibrotic HP.

**Methods:**

The incidence rates of AE were calculated in 101 patients with biopsy-proven HP. AE was defined as the worsening of dyspnoea within 30 days, with new bilateral lung infiltration and no evidence of infection or other causes of dyspnoea.

**Results:**

During follow-up (median: 30 months), 18 (17.8%) patients experienced AE. The 1, 3, and 5 year incidence rates of AE were 6.0, 13.6, and 22.8%, respectively. Lower diffusing capacity of the lung for carbon monoxide (DL_CO_) and a radiologic usual interstitial pneumonia (UIP)-like pattern were risk factors for AE. In-hospital mortality after AE was 44.4%. Median survival from diagnosis was significantly shorter in patients with AE (26.0 months) than in those with no-AE RD (55.0 months; *p* = 0.008) or no RD (not reached; *p* < 0.001). AE remained a significant predictor of all-cause mortality (hazard ratio, 8.641; 95% confidence interval, 3.388–22.040; p < 0.001) after adjustment for age, body mass index, lung function, lymphocyte levels in bronchoalveolar lavage fluid, and the presence of a UIP-like pattern.

**Conclusions:**

AE was not uncommon among patients with fibrotic HP and significantly affected prognosis. A lower DL_CO_ value and radiologic UIP-like pattern at diagnosis were associated with the development AE in patients with fibrotic HP.

**Supplementary Information:**

The online version contains supplementary material available at 10.1186/s12931-021-01748-2.

## Introduction

Hypersensitivity pneumonitis (HP) is an interstitial lung disease (ILD) that results from inhalation of organic antigens in susceptible individuals [1]. According to the recently published guidelines for diagnosis of hypersensitivity pneumonitis, HP is classified into fibrotic or nonfibrotic [2], since the presence of radiologic or histopathologic fibrosis is important in determining prognosis [3–5]. The progressive fibrosing nature of fibrotic HP is also characteristic of idiopathic pulmonary fibrosis (IPF), a prototype of fibrosing ILD [6], and the radiologic pattern of usual interstitial pneumonia (UIP) is often indistinguishable between cases of fibrotic HP and IPF [7]. Furthermore, genomic risk factors such as the MUC5B promotor polymorphism and shorter telomere lengths, known to be associated with the development and progression of IPF, are also associated with the extent of fibrosis in HP [8].

In IPF, the development of acute exacerbation (AE) is a well-known risk factor for a poor outcome, with an in-hospital mortality rate of approximately 50% [9–12]. It has been noted that AE can occur not only in IPF but also in other fibrosing ILDs, including HP [13–18]. However, only few studies have examined AE in HP. In 2008, Olson et al. reported a case series on AE in chronic HP [15]. Four patients developed AE, which resulted in respiratory failure requiring mechanical ventilation; three of them died and one had to undergo lung transplantation. In their retrospective study involving 100 patients with chronic bird fancier’s lung, Miyazaki et al. also found a high mortality rate following AE (12 of 14 patients, 85.7%) [18]. Although these results demonstrate detrimental outcomes, it remains challenging to determine how AE affects patient prognosis in fibrotic HP owing to the limited amount of data and knowledge on the incidence and risk factors of AE [15, 18]. Therefore, in this study, we aimed to determine the incidence rate and risk factors of AE and its effect on the survival of patients with fibrotic HP.

## Methods

### Study patients

A total of 101 patients diagnosed with definite HP based on histopathological findings (surgical lung biopsy: 99, transbronchial lung biopsy: 2) were identified between January 2002 and December 2017 at Asan Medical Center, Seoul, Republic of Korea. All patients showed radiologic and/or histopathological fibrosis. Their exposure history to causative antigens is shown in Table S1 in Additional file 1. All diagnoses were made through multidisciplinary discussions. The study protocol was approved by the Institutional Review Board of Asan Medical Center (IRB No.: 2017-1081). The requirement for informed consent was waived due to the retrospective nature of the study.

### Clinical data

Clinical and survival data of all patients were retrospectively collected from medical records, telephone interviews, and/or records from the National Health Insurance of Korea. Spirometry was performed, and the diffusing capacity of the lung for carbon monoxide (DL_CO_) and total lung capacity (TLC) were measured according to the ATS/European Respiratory Society recommendations [19–21]. Results are expressed as percentages of the normal predicted values. The 6-min-walk test and bronchoalveolar lavage (BAL) were performed in accordance with previously published guidelines [22, 23].

Records of follow-up visits, which usually occurred every 3–6 months, and hospitalisation data were reviewed to identify the development of complications such as pneumonia, AE, pneumothorax, and pulmonary hypertension. Rapid deterioration (RD) was defined as an acute worsening of dyspnoea, requiring hospitalisation and presenting with new radiologic abnormalities [9]. RD included AE as well as other acute respiratory events including pneumonia or pneumothorax. AE was defined, using the criteria previously used in IPF by Collard et al. (2007), as a worsening of dyspnoea within 30 days, with new bilateral lung infiltration and no evidence of infection or other alternative causes of dyspnoea (e.g. pulmonary embolism and left heart failure) [24]. AE was labelled as ‘suspected AE’ when the aetiology of acute respiratory worsening was unknown but did not fulfil all the AE criteria owing to missing data. When infection was strongly suspected clinically (symptoms such as purulent sputum and rapid improvement and response to antibiotic treatment alone) but causative organism was not identified, it was categorised as ‘suspected infection’.

### Radiologic assessment

High-resolution computed tomography (HRCT) images of all patients were evaluated by two thoracic radiologists (J.C., E.J.C.) who were blinded to the clinical data. Based on HRCT findings, patients were classified as having a UIP-like pattern or not. Discrepancies were resolved by a consensus. A UIP-like pattern was diagnosed when HRCT findings were compatible with a UIP or probable UIP pattern according to the HRCT classification of Fleischner Society IPF diagnostic guidelines with modification [25]. Briefly, a UIP-like pattern was defined as a reticular pattern with traction bronchiectasis or bronchiolectasis with/without honeycombing. Given that mosaic attenuation is frequently observed in HP [26] and distribution of fibrosis in HP may differ from that in IPF [27], the presence of mosaic attenuation and distribution of fibrosis were not considered as features of alternative diagnosis in this study [28].

### Statistical analysis

Data are presented as mean ± standard deviation or median [interquartile range] for continuous variables and percentages for categorical variables. The Student’s t-test or Mann–Whitney test was used for continuous variables, and the chi-squared test and Fisher’s exact test were used to compare categorical variables. Kaplan–Meier estimates and the log-rank test were used for survival analysis. Cumulative incidence rates of RD and AE were also estimated using Kaplan–Meier survival analysis. Risk factors for RD and AE, and predictors of all-cause mortality were analysed using a Cox proportional hazards analysis. Variables with p < 0.05 in the unadjusted analysis were entered into multivariable models. Logistic regression analysis was used to determine risk factors for in-hospital mortality. All p-values were two-tailed, and p-values < 0.05 were considered statistically significant. Data were analysed using the Statistical Package for the Social Sciences software version 23.0 (IBM Corp., Armonk, N.Y., USA).

## Results

### Incidence

**Fig. 1:**
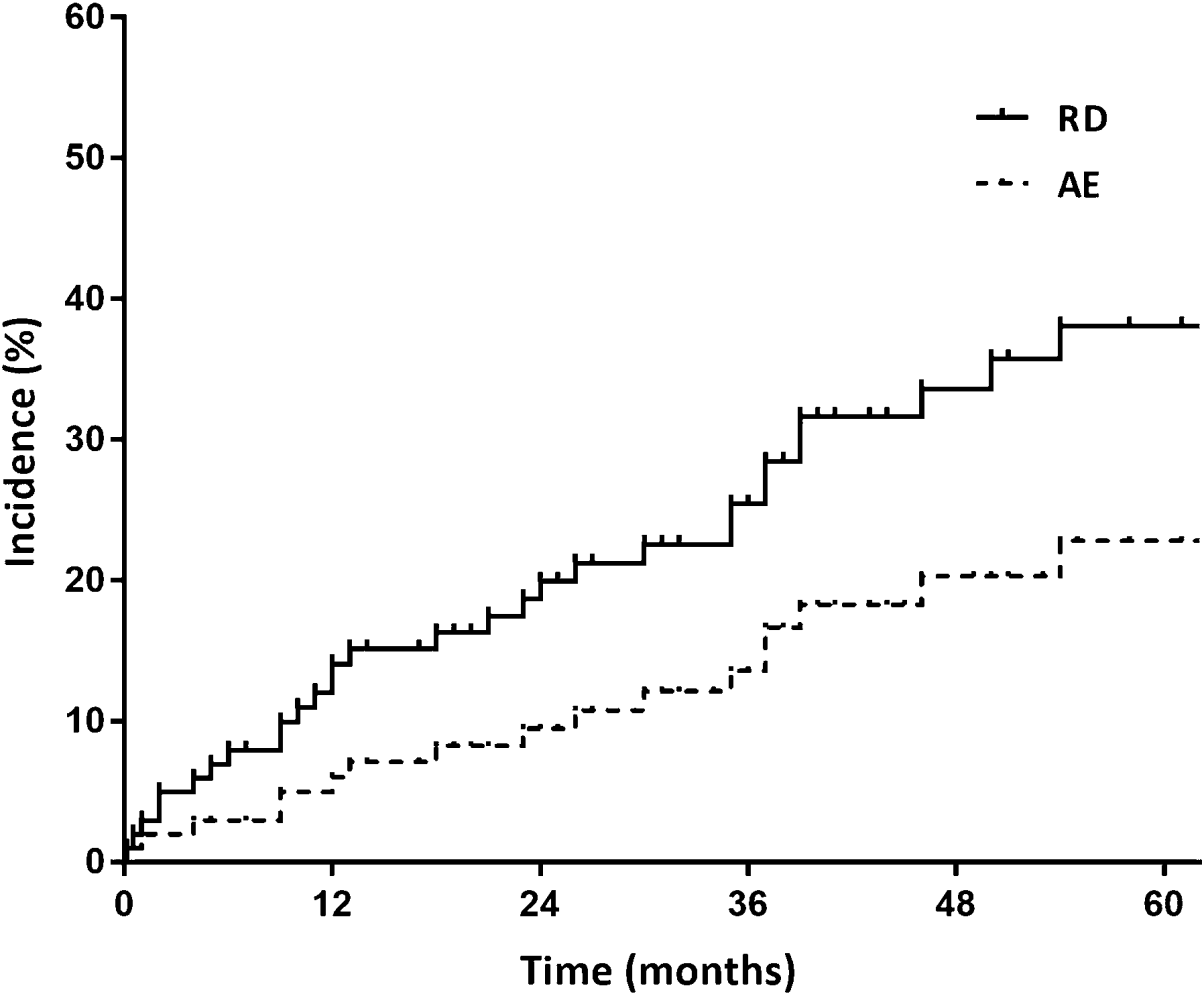
The 1, 3, and 5 year cumulative incidence rates of RD and AE in patients with fibrotic HP. *AE* acute exacerbation, *HP* hypersensitivity pneumonitis, *RD* rapid deterioration

The median follow-up duration was 30.0 months (interquartile range, 15.0–51.0 months). During follow-up, 33 patients (32.7%) experienced RD. The 1-, 3-, and 5-year cumulative incidence rates of RD were 14.0%, 25.4%, and 38.0%, respectively (Fig. 1). The aetiologies of RD are shown in Table 1. AE was the most common cause of RD (54.5%), followed by infection (36.4%). The 1, 3, and 5 year cumulative incidence rates of AE were 6.0, 13.6, and 22.8%, respectively (Fig. 1).

**Table 1:** Aetiologies of rapid deterioration

Aetiology	Cases	Documented organisms (n)
Total RD	33 (32.7)	
Bilateral lesion		
Acute exacerbation	18 (17.8)	
Definite	9 (8.9)	
Suspected	9 (8.9)	
Infection	9 (8.9)	
Viral	4 (4.0)	Influenza (2), Respiratory syncytial virus (2)
Mycobacterial	1 (1.0)	Mycobacterium tuberculosis (1)
Suspected	4 (4.0)	
Focal lesion		
Pneumothorax	3 (3.0)	
Infection	3 (5.0)	
Bacterial	1 (1.0)	Pseudomonas aeruginosa (1)
Mycobacterial	1 (1.0)	Mycobacterium tuberculosis (1)
Suspected	1 (1.0)	

Data are presented as n (%). Aetiologies of the first episode of rapid deterioration are shown

### Risk factors for RD and AE

The mean age of all patients was 58.9 years, and 60.4% were female. Patients who developed RD had a significantly lower forced vital capacity (FVC), DL_CO_, and TLC and a shorter 6-min-walk distance (6MWD) at diagnosis than did those with no RD (Table 2). A significantly lower DL_CO_, shorter 6MWD, and greater number of patients with a UIP-like pattern were found among patients with AE than among those with no RD. In addition, the proportion of patients with a UIP-like pattern on HRCT was significantly higher in patients with AE than in those with no-AE RD.

**Table 2:** Comparison of baseline characteristics between RD and no-RD groups among patients with fibrotic HP

Characteristics	RD			No RD
	Total	AE	No AE	
Patients	33	18	15	68
Age, years	65.0 [54.5;70.0]^†^	62.5 [53.5;70.3]	59.0 [54.0;66.0]	62.0 [50.5;67.0]
Female sex	21 (63.6)	10 (55.6)	11 (73.3)	40 (58.8)
BMI, kg/m^2^	23.6 [20.4;26.8]^†^	24.3 [21.9;26.8]	25.5 [23.4;28.0]	24.4 [22.7;27.1]
Smoking				
Ever smoker	11 (33.3)	7 (38.9)	4 (26.7)	27 (39.7)
Smoking amount, pack-years	30.0 [15.0;35.0]	20.0 [15.0;30.0]	35.0 [9.1;35.0]	30 [12.5;45.0]
Positive history of exposure to antigen	27 (81.8)	15 (83.3)	12 (80.0)	58 (85.3)
Pulmonary function test (%pred.)				
FVC	63.8 ± 21.9^†^	67.7 ± 23.4	59.2 ± 19.6^†^	74.8 ± 14.0
DL_CO_	50.8 ± 18.3^†^	50.5 ± 21.7^†^	51.2 ± 14.3^†^	64.6 ± 14.4
TLC	68.0 ± 16.6^†^	71.9 ± 18.9	63.9 ± 13.3^†^	76.2 ± 10.8
6 min-walk test				
Distance, m	405.0 [330.0;476.5]^†^	400.0 [287.5;476.3]	460.0 [404.0;490.0]	460.0 [405.0;500.0]
Lowest saturation, %	91.0 [87.5;94.0]^†^	90.0 [87.8;93.3]	92.0 [88.0;95.0]	93.0 [90.0;95.0]
BALF analysis				
Total WBC, /μL	205.0 [130.0;335.0]	180.0 [132.5;252.5]	120.0 [80.0;275.0]	180.0 [115.0;340.0]
Neutrophil, %	4.0 [1.3;6.8]	4.0 [1.0;6.8]	5.0 [1.0;10.0]	4.0 [1.5;6.5]
Lymphocyte, %	18.0 [13.0;47.0]	17.5 [8.5;30.5]	31.0 [15.0;59.5]	18.0 [11.0;32.5]
Lympho-dominance (lymphocyte > 20%)	11 (33.3)	5 (27.8)	6 (40.0)	31 (45.6)
UIP-like pattern on HRCT	20 (60.6)	15 (83.3)*^,^^†^	5 (33.3)	36 (52.9)
Treatment				
No treatment	0 (0.0)	0 (0.0)	0 (0.0)	10 (14.7)
Corticosteroid ± immunosuppressants	33 (100.0)	18 (100.0)	15 (100.0)	58 (85.3)

Data are presented as mean ± standard deviation, median [interquartile range], or n (%)

*AE* acute exacerbation, *BALF* bronchoalveolar lavage fluid, *BMI* body mass index, *DL*_*CO*_ diffusing capacity of the lung for carbon monoxide, *FVC* forced vital capacity, *HP* hypersensitivity pneumonitis, *HRCT* high-resolution computed tomography, *% pred.* % of the predicted value, *RD* rapid deterioration, *TLC* total lung capacity, *UIP* usual interstitial pneumonia, *WBC* white blood cell

*p < 0.05 vs no AE

^†^p < 0.05 vs no RD

To identify factors significantly associated with the development of AE and RD, Cox proportional hazards analyses were performed. A lower DL_CO_ and the presence of a UIP-like pattern were identified as significant risk factors for AE both in the unadjusted and multivariable analyses (Table 3). Regarding RD, older age and lower DL_CO_, were identified as significant risk factors in the multivariable analysis (Table S2 in Additional file 1).

**Table 3:** Risk factors for AE in patients with fibrotic HP

Characteristics	Unadjusted model		Multivariable model	
	HR (95% CI)	*p*-value	HR (95% CI)	*p*-value
Age	1.038 (0.988–1.091)	0.139		
Female sex	1.303 (0.514–3.306)	0.577		
BMI	0.919 (0.810–1.042)	0.188		
Ever smoker	1.001 (0.386–2.591)	0.999		
Positive history of exposure to antigen	1.239 (0.355–4.317)	0.737		
Pulmonary function test				
FVC	0.987 (0.955–1.007)	0.151		
DL_CO_	0.959 (0.934–0.985)	0.002	0.960 (0.935 – 0.985)	0.002
TLC	0.980 (0.944–1.017)	0.291		
BALF analysis				
Neutrophil count	0.984 (0.909–1.065)	0.689		
Lymphocyte count	0.970 (0.937–1.003)	0.072		
UIP-like pattern on HRCT	5.481 (1.566–19.182)	0.008	4.013 (1.128 – 14.283)	0.032

*AE* acute exacerbation, *BALF* bronchoalveolar lavage fluid, *BMI* body mass index, *DL*_*CO*_ diffusing capacity of the lung for carbon monoxide, *FVC* forced vital capacity, *HP* hypersensitivity pneumonitis, *HR* hazard ratio, *HRCT* high-resolution computed tomography, *TLC* total lung capacity, *UIP* usual interstitial pneumonia

### Effect on overall survival

**Fig. 2:**
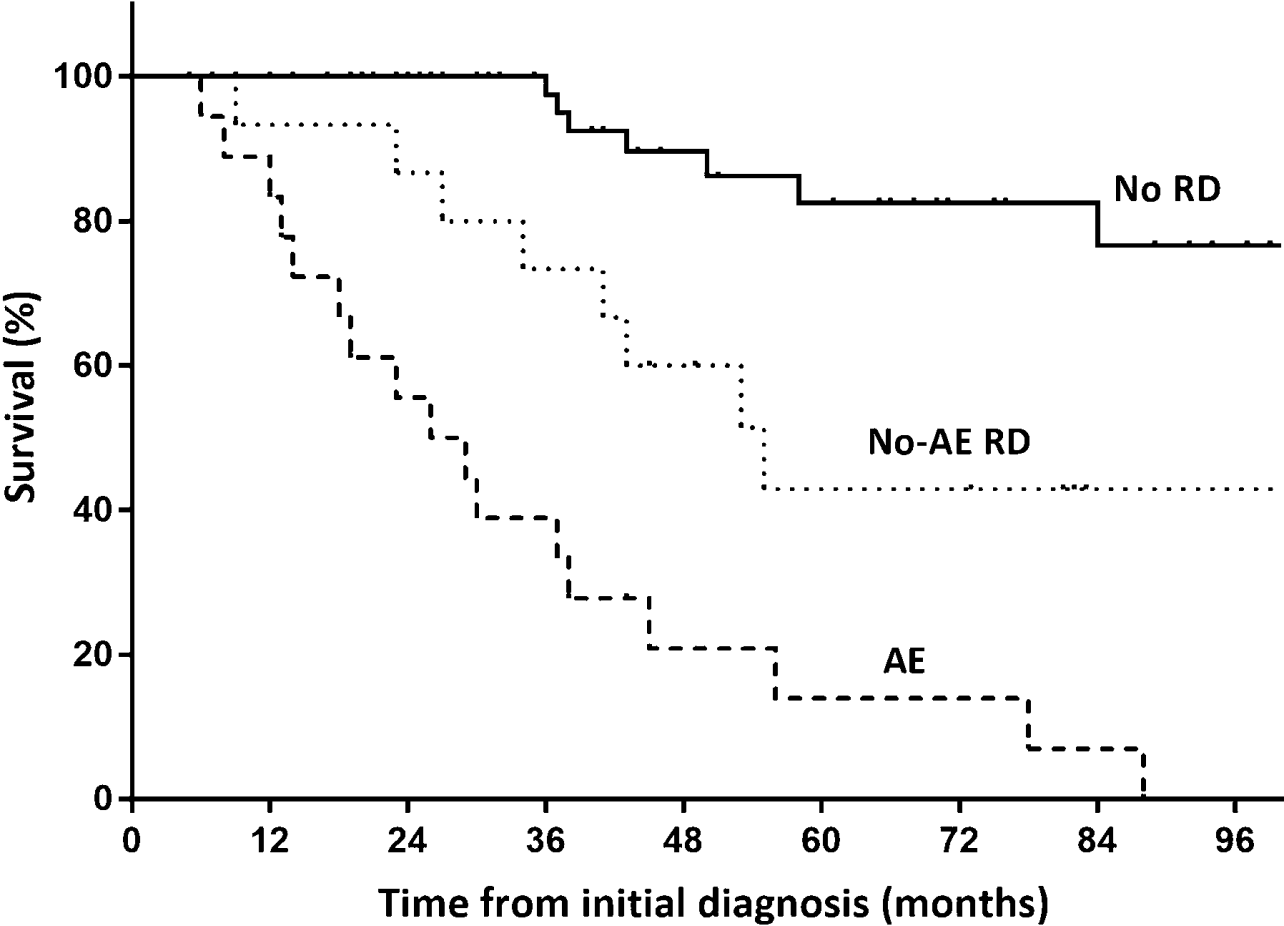
Survival from the initial diagnosis in patients with AE, no-AE RD, and no RD. Patients with AE showed a significantly poorer survival than those with no-AE RD or with no RD. *AE* acute exacerbation, *RD* rapid deterioration

Patients who experienced RD, with or without AE, showed a significantly poorer survival following diagnosis than did those without RD (Fig. 2). The median survival of patients with no-AE RD and AE was 55.0 months (95% confidence interval [CI], 36.3–73.7 months) and 26.0 months (95% CI, 13.5–38.5 months), respectively. Their median overall survival was significantly shorter than that of patients with no RD (median survival not reached; *p* = 0.013 and *p* < 0.001, respectively). Among the patients with RD, those with AE had a significantly shorter survival than those with no-AE RD (*p* = 0.008).

The predictors of all-cause mortality are shown in Table 4. In the unadjusted analysis, the factors found to be significantly associated with mortality were older age; a lower BMI, FVC, DL_CO_, and TLC; lower lymphocyte levels in BAL fluid; the presence of a UIP-like pattern; and the development of AE. The development of AE remained as a significant predictor even after adjustment for other variables, and it appeared to exert the greatest effect on mortality risk (hazard ratio, 8.641; 95% CI, 3.388–22.040; *p* < 0.001).

**Table 4:** Predictors of all-cause mortality in patients with fibrotic HP

Characteristics	Unadjusted model		Multivariable model	
	HR (95% CI)	*p*-value	HR (95% CI)	*p*-value
Age	1.043 (1.006–1.082)	0.023	1.105 (1.040–1.174)	0.001
Female sex	1.129 (0.558–2.285)	0.735		
BMI	0.899 (0.815–0.992)	0.035	0.859 (0.738–1.000)	0.050
Ever smoker	0.767 (0.371–1.583)	0.472		
Positive history of exposure to antigen	1.295 (0.531–3.156)	0.570		
Pulmonary function test				
FVC	0.973 (0.954–0.993)	0.008	1.011 (0.976–1.047)	0.548
DL_CO_	0.956 (0.934–0.978)	< 0.001	0.960 (0.914–1.009)	0.108
TLC	0.958 (0.931–0.986)	0.003		
BALF analysis				
Neutrophil count	0.977 (0.917–1.041)	0.478		
Lymphocyte count	0.977 (0.956–1.000)	0.046	0.973 (0.946–1.001)	0.061
UIP-like pattern on HRCT	2.519 (1.211–5.241)	0.013	0.878 (0.276–2.791)	0.825
Acute exacerbation	9.825 (4.846–19.917)	< 0.001	8.641 (3.388–22.040)	< 0.001

*BALF* bronchoalveolar lavage fluid, *BMI* body mass index, *DL*_*CO*_ diffusing capacity of the lung for carbon monoxide, *FVC* forced vital capacity, *HP* hypersensitivity pneumonitis, *HR* hazard ratio; *HRCT* high-resolution computed tomography, *TLC* total lung capacity, *UIP* usual interstitial pneumonia

We did not include TLC in the multivariable model, as it strongly correlated with FVC (correlation coefficient, r = 0.873; *p* < 0.001)

### Survival after hospitalisation

Clinical characteristics at the time of hospitalisation were compared between patients with AE and those with bilateral infection to identify factors that could help distinguish between them (Table S3 in Additional file 1). The total white blood cell (WBC) count and percentage of neutrophils in the BAL fluid were significantly higher in patients with bilateral infection than in those with AE (WBC: 8066.0/µL vs 206.4/µL, *p* = 0.011; neutrophils: 50.4 vs 13.4%, *p* = 0.019). However, there was no significant difference in age, sex, disease duration, fever, level of C-reactive protein, and lung function.

**Fig. 3:**
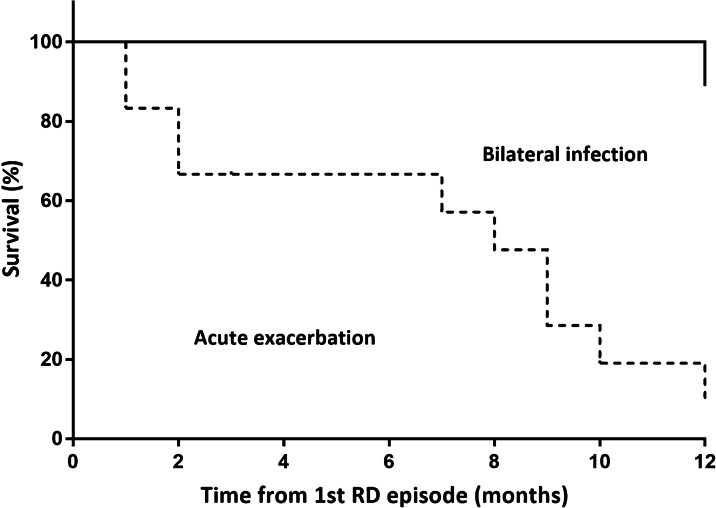
Survival following the development of AE and bilateral infection. Patients with AE showed a significantly poorer survival than did those with bilateral infection. *AE* acute exacerbation

The mortality outcomes following the hospitalisation of patients with AE and those with bilateral infection are shown in Fig. 3. In-hospital mortality after AE was 44.4% (8/18), whereas none of the patients with bilateral infection died. Patients who experienced AE showed significantly poorer outcomes; the 30 day, 90 day, and 1 year mortality were significantly higher in patients with AE (33.3, 55.6, 83.3%, respectively) than in those with bilateral infection (no death; all *p*-values < 0.05).

Clinical characteristics of survivors and non-survivors of AE and their treatment regimens are illustrated in Tables S4 and S5, respectively, in Additional file 1. There was no significant difference in the treatment regimens between survivors and non-survivors including the use of cytotoxic agents. A significantly lower partial pressure of oxygen to fraction of inspired oxygen ratio measured at the time of hospitalisation was found to be significantly associated with in-hospital mortality following AE (odds ratio, 0.983; 95% CI, 0.967–0.999; *p* = 0.040), as shown in Table 5.

**Table 5:** Risk factors for in-hospital mortality following AE in patients with fibrotic HP

Characteristics	Unadjusted model	
	Odds ratio (95% CI)	*P*-value
Age	0.998 (0.922–1.081)	0.964
Female sex	4.500 (0.585–34.608)	0.148
BMI	0.969 (0.763–1.230)	0.793
Ever smoker	0.333 (0.044–2.523)	0.287
Fever	0.500 (0.065–3.845)	0.505
Purulent sputum	5.400 (0.437–66.671)	0.188
PaO_2_/FiO_2_	0.983 (0.967–0.999)	0.040
CRP	1.070 (0.959–1.194)	0.229
FVC^*^	0.982 (0.935–1.032)	0.474
DL_CO_^*^	0.983 (0.931–1.038)	0.537

Presented data are the values at the time of AE

*AE* acute exacerbation, *BMI* body mass index, *CI* confidence interval, *CRP* C-reactive protein, *DL*_*CO*_ diffusing capacity of the lung for carbon monoxide, *FiO*_*2*_ fraction of inspired oxygen, *FVC* forced vital capacity, *HP* hypersensitivity pneumonitis, *PaO2* Partial pressure of oxygen

*Presented data are closest measured values before AE (median interval: 3.0 months)

## Discussion

In this study, we evaluated the incidence, risk factors, and outcomes of AE in patients with fibrotic HP. The 1, 3, and 5 year cumulative incidence rates of AE were 6.0, 13.6, and 22.8%, respectively. A lower DL_CO_ and the presence of a UIP-like pattern were significant risk factors for AE. AE was associated with a poor prognosis and high in-hospital mortality rate (44.4%), and it appeared to have the greatest effect on overall survival.

As previous studies have shown, AE occurs not only in IPF but also in non-IPF fibrosing ILD [14–18, 29]. According to a recent retrospective study by Suzuki et al. that included 462 IPF and 557 non-IPF ILD patients, AE had a significant effect on mortality, regardless of diagnosis [30]. The 90- ay mortality rate following AE was similar between IPF and non-IPF ILD (47 and 38%, *p* = 0.345). However, patients with HP comprised only 5.2% of the non-IPF ILD patients in that study, thus limiting the generalisability of the result in the HP population. There is also lack of data regarding the incidence of AE in HP. In the aforementioned study by Suzuki et al., the incidence rate of AE in HP was 6.05 per 100 patient-years and that in IPF was 8.38 per 100 patient-years [30]. Considering the results of a previous study on IPF patients conducted at our institution [9], the cumulative incidence rates of AE seem to be lower in patients with fibrotic HP than in those with IPF (1 and 3 year incidence rates were 6.0 and 13.6%, respectively, in fibrotic HP; 14.2 and 20.7%, respectively, in IPF). To accurately estimate the incidence rate of AE in fibrotic HP, further prospective studies are needed.

In this study, a lower DL_CO_ and the presence of a UIP-like pattern on HRCT at diagnosis were risk factors for AE. This result is in line with a previous report. In a retrospective study involving 100 patients with bird fancier’s lung, patients who developed AE had a lower TLC and DL_CO_ at baseline than did those without AE [18]. In IPF, lower lung function is also known as a risk factor for AE [9, 31–33]; it is likely that patients with advanced disease are at a greater risk of AE, regardless of the underlying pathology. A UIP-like pattern on HRCT was another significant risk factor for AE. In the aforementioned study evaluating patients with chronic bird fancier’s lung, patients with UIP-like lesions on surgical lung biopsy experienced AE more frequently. No other studies have evaluated whether a radiologic UIP-like pattern is associated with AE in HP. However, a UIP pattern on HRCT was associated with a higher risk of AE in non-IPF ILDs such as rheumatoid arthritis-associated ILD [16].

The high in-hospital mortality rate following AE in our study, which was almost similar to that of IPF, should be noted [9, 11]. Interestingly, there was a significant difference in the outcomes of AE and bilateral infection. With regard to IPF, findings suggest that the outcome is similar in idiopathic and triggered AE [6, 9, 34]. Thus, the previous definition of AE in IPF, which is limited to idiopathic acute respiratory worsening, was revised to include all events—both idiopathic and triggered—characterised by acute lung injury [34]. On the contrary, when we classified our patients based on whether they had AE (idiopathic worsening) or bilateral infection, patients with infection showed a better outcome than did those with AE. So far, the definition of AE in IPF has been widely adopted in previous studies on non-IPF ILD [13, 16]; given that our study demonstrates a difference between the outcomes of idiopathic worsening (AE) and infection, the cause of acute respiratory worsening may be important in predicting outcomes in fibrotic HP. However, the interpretation should be cautious because our analysis included only small number of patients. Further studies are required to evaluate this issue.

This study has some limitations First, this was a retrospective study conducted at a single tertiary hospital. However, in our study, the clinical characteristics of patients were similar to those of other studies evaluating patients with HP [18, 35]. Second, the study patients were collected over 15 years during which diagnostic approach might have changed. To ensure the diagnosis, we included patients with biopsy confirmed HP. One may argue this might have resulted in including only patients with atypical features who therefore required a histological confirmation of their diagnosis. However, clinical features highly suggestive of HP were fairly frequently found among study patients (e.g., history of antigen exposure and lympho-dominance in BAL fluid in 85.1% and 41.6% of the patients, respectively). Third, BAL or endotracheal aspiration was not performed in all patients with RD; therefore, the distinction between AE and infection may not be perfect. In some patients, BAL could not be performed due to instability, but microbial tests, including respiratory virus PCR panels, were performed for all patients. Our diagnostic approach reflects the real clinical practice in which the differential diagnosis between AE and infection is often difficult. Finally, the definition of a UIP-like pattern used in this study was modified from the HRCT classification of IPF diagnostic guidelines to include a feature inconsistent with UIP (e.g. mosaic attenuation). In the absence of a consensus on the definition of the UIP-like pattern in fibrosing ILDs other than IPF, the modification may be inevitable in some subtypes of ILDs, as previously described [28, 36]. In a recent randomised controlled trial of progressive fibrosing ILD, the presence of mosaic attenuation or centrilobular nodules was also accepted. Furthermore, when categorised using the definition, patients with a UIP-like pattern tended to have a more rapid FVC decline [36], suggesting the usefulness of this definition.

In conclusion, AE was not uncommon in patients with fibrotic HP and, similar to IPF, exerted a significant effect on prognosis. A lower DL_CO_ and the presence of a UIP-like pattern on HRCT at diagnosis were associated with AE in patients with fibrotic HP.

## Supplementary Information

**Additional file 1.** Additional tables.

## Data Availability

The datasets used and/or analysed during the current study are available from the corresponding author on reasonable request.

## Abbreviations

6MWD: 6-Minute walk distance
AE: Acute exacerbation
ATS: American Thoracic Society
BAL: Bronchoalveolar lavage
CI: Confidence interval
DLCO: Diffusing capacity of the lung for carbon monoxide
FVC: Forced vital capacity
HP: Hypersensitivity pneumonitis
HRCT: High-resolution computed tomography
ILD: Interstitial lung disease
IPF: Idiopathic pulmonary fibrosis
RD: Rapid deterioration
TLC: Total lung capacity
UIP: Usual interstitial pneumonia
WBC: White blood cell
