# Supplementary material for: Acute exacerbation of fibrotic hypersensitivity pneumonitis: incidence and outcomes
Source: Respir Res. 2021 May 20;22:152. doi: 10.1186/s12931-021-01748-2 (PMC8138994; doi:10.1186/s12931-021-01748-2)
Supplement: Supplementary file 1 — Additional file 1. Additional tables. [file 12931_2021_1748_MOESM1_ESM.docx]

Additional File 1

Acute exacerbation of fibrotic hypersensitivity pneumonitis: incidence and outcomes

Jieun Kang^1,2^, Yeon Joo Kim^1^, Jooae Choe^3^, Eun Jin Chae^3^, Jin Woo Song^1^

^1^Department of Pulmonary and Critical Care Medicine, Asan Medical Center, University of Ulsan College of Medicine, Seoul, Republic of Korea

^2^Division of Pulmonary and Critical Care Medicine, Department of Internal Medicine, Ilsan Paik Hospital, Inje University College of medicine, Ilsan, Republic of Korea

^3^Department of Radiology and Research Institute of Radiology, Asan Medical Center, University of Ulsan College of Medicine, Seoul, Republic of Korea

Supplementary Table 1. Exposure history to causative antigens in the study patients

| Characteristics | Total |
| --- | --- |
| Patients, number | 101 |
| Microbes (mold, humid hay, cotton) | 50 (49.5) |
| Chemical (insecticides, cement, dye, fertilizers) | 18 (17.8) |
| Animal protein (birds’ feather) | 8 (7.9) |
| Enzymes (mushrooms) | 1 (1.0) |
| Metal (fumes) | 1 (1.0) |
| Pharmaceutical agents (drugs) | 1 (1.0) |
| Plant proteins (flour, vegetable processing) | 7 (6.9) |
| Unidentifiable antigen | 15 (14.9) |

Data are presented as number (%), unless otherwise indicated.

Supplementary Table 2. Risk factors for RD in patients with fibrotic HP

| Characteristics | Unadjusted model | | Multivariable model | |
| --- | --- | --- | --- | --- |
|  | HR (95% CI) | p-value | HR (95% CI) | p-value |
| Age | 1.054 (1.013–1.096) | 0.009 | 1.072 (1.029–1.116) | 0.001 |
| Female sex | 1.101 (0.541–2.239) | 0.791 |  |  |
| BMI | 0.915 (0.830–1.007) | 0.070 |  |  |
| Ever smoker | 0.752 (0.363–1.556) | 0.442 |  |  |
| Positive history of exposure to antigen | 1.488 (0.607–3.644) | 0.385 |  |  |
| Pulmonary function test |  |  |  |  |
| FVC | 0.960 (0.940–0.980) | <0.001 | 0.984 (0.958–1.011_ | 0.239 |
| DL_CO_ | 0.959 (0.941–0.977) | <0.001 | 0.941 (0.908–0.975) | 0.001 |
| TLC | 0.951 (0.926–0.977) | <0.001 |  |  |
| BALF analysis |  |  |  |  |
| Neutrophil count | 0.994 (0.943–1.047) | 0.812 |  |  |
| Lymphocyte count | 0.995 (0.976–1.014) | 0.587 |  |  |
| UIP-like pattern on HRCT | 1.664 (0.812–3.412) | 0.165 |  |  |

BALF, bronchoalveolar lavage fluid; BMI, body mass index; DL_CO_, diffusing capacity of the lung for carbon monoxide; FVC, forced vital capacity; HP, hypersensitivity pneumonitis; HR, hazard ratio; HRCT, high-resolution computed tomography; RD, rapid deterioration; TLC, total lung capacity; UIP, usual interstitial pneumonia.

We did not include TLC in the multivariable model, as it strongly correlated with FVC (correlation coefficient, r = 0.873; p < 0.001).

Supplementary Table 3. Comparison of clinical characteristics at the time of hospitalisation between patients with AE and bilateral infection

| Characteristics | AE | Infection | p-value |
| --- | --- | --- | --- |
| Number of patients | 18 | 9 |  |
| Age, years | 65.5 ± 9.0 | 61.6 ± 19.5 | 0.955 |
| Female sex | 10 (55.6) | 7 (77.8) | 0.406 |
| BMI, kg/m^2^ | 23.8 ± 4.0 | 25.4 ± 8.0 | 0.503 |
| Ever smoker | 7 (38.9) | 3 (33.3) | >0.999 |
| Disease duration, months | 33.5 ± 24.0 | 59.3 ± 36.5 | 0.156 |
| Fever | 6 (33.3) | 4 (44.4) | 0.683 |
| PaO_2_/FiO_2_ | 253 ± 83 | 228 ± 91 | 0.259 |
| CRP, mg/dL | 7.8 ± 8.9 | 10.0 ± 12.5 | 0.865 |
| FVC^*^, % pred | 58.6 ± 20.1 | 57.4 ± 17.1 | 0.883 |
| DL_CO_^*^, % pred | 42.9 ± 18.5 | 52.9 ± 16.3 | 0.186 |
| BALF^†^ |  |  |  |
| Total WBC, /μL | 206.4 ± 105.9 | 8066.0 ± 13547.6 | 0.011 |
| Neutrophil, % | 13.4 ± 15.9 | 50.4 ± 38.8 | 0.019 |
| Lymphocyte, % | 24.4 ± 18.5 | 14.8 ± 25.4 | 0.126 |

Data are presented as mean ± standard deviation or n (%).

AE, acute exacerbation; BALF, bronchoalveolar lavage fluid; BMI; body mass index; CRP, C-reactive protein; DL_CO_, diffusing capacity of the lung for carbon monoxide; FVC, forced vital capacity; % pred., % of the predicted value; WBC, white blood cell.

^*^Presented data are closest measured values before AE or infection (median interval: 2.0 months)

^†^BAL was performed in 9 and 5 of the patients with AE and infection, respectively. The presented data are from the bronchoalveolar lavage performed at AE or infection.

Supplementary Table 4. Comparison of clinical characteristics of patients with AE at the time of hospitalisation according to their in-hospital mortality outcomes

|  | Survivors | Non-survivors | p-value |
| --- | --- | --- | --- |
| Number of patients | 10 | 8 |  |
| Age, years | 63.0 ± 11.7 | 62.8 ± 13.2 | 0.790 |
| Female sex | 4 (40.0) | 6 (75.0) | 0.188 |
| BMI, kg/m^2^ | 24.0 ± 3.7 | 23.5 ± 4.6 | 0.859 |
| Ever smoker | 5 (50.0) | 2 (25.0) | 0.367 |
| Fever | 4 (40.0) | 2 (25.0) | 0.638 |
| Purulent sputum | 1 (10.0) | 3 (37.5) | 0.275 |
| PaO_2_/FiO_2_ | 314.6 ± 114.2 | 179.0 ± 63.6 | 0.012 |
| CRP, mg/dL | 5.6 ± 9.7 | 11.2 ± 9.2 | 0.248 |
| FVC^*^, %pred. | 61.6 ± 20.7 | 54.9 ± 20.2 | 0.374 |
| DL_CO_^*^, %pred. | 45.4 ± 19.7 | 40.0 ± 18.0 | 0.531 |

Data are presented as mean ± standard deviation or n (%).

^*^Presented data are values measured closest before AE (median interval: 3.0 months)

AE, acute exacerbation; BMI, body mass index; CRP, c-reactive protein; DL_CO_, diffusing capacity of the lung for carbon monoxide; FVC, forced vital capacity; % pred., % of the predicted value.

Supplementary Table 5. Types of treatment for acute exacerbation

|  | Survivors | Non-survivors | p-value |
| --- | --- | --- | --- |
| Number of patients | 10 | 8 | 0.245 |
| Steroid pulse^*^ | 2 (20.0) | 2 (25.0) |  |
| Steroid pulse + cytotoxic agent^†^ | 0 (0.0) | 2 (25.0) |  |
| High-dose steroid^‡^ | 7 (70.0) | 3 (37.5) |  |
| High-dose steroid + cytotoxic agent | 0 (0.0) | 0 (0.0) |  |
| Low-dose steroid^§^ | 1 (10.0) | 0 (0.0) |  |
| Low-dose steroid + cytotoxic agent | 0 (0.0) | 1 (12.5) |  |

^*^steroid pulse therapy was defined as methylprednisolone > 500 mg for 3 days followed by high-dose steroid administration.

^†^cytotoxic agent was azathioprine, cyclosporine, or cyclophosphamide.

^‡^high-dose steroid therapy was defined as prednisolone (or equivalent dose) > 0.5mg/kg/day.

^§^low-dose steroid therapy was defined as prednisolone (or equivalent dose) ≤ 0.5mg/kg/day.
